# Breast fibroblasts modulate epithelial cell proliferation in three-dimensional *in vitro *co-culture

**DOI:** 10.1186/bcr949

**Published:** 2004-11-08

**Authors:** Andrea Sadlonova, Zdenek Novak, Martin R Johnson, Damon B Bowe, Sandra R Gault, Grier P Page, Jaideep V Thottassery, Danny R Welch, Andra R Frost

**Affiliations:** 1 Department of Pathology, The University of Alabama at Birmingham, Alabama, USA; 2 Department of Pediatrics, The University of Alabama at Birmingham, Alabama, USA; 3 Department of Pharmacology and Toxicology, The University of Alabama at Birmingham, Alabama, USA; 4 Department of Biostatistics, The University of Alabama at Birmingham, Alabama, USA; 5 Drug Discovery Division, Southern Research Institute, Birmingham, Alabama, USA

**Keywords:** breast cancer, breast epithelium, fibroblast, insulin-like growth factor, stroma

## Abstract

**Background:**

Stromal fibroblasts associated with *in situ *and invasive breast carcinoma differ phenotypically from fibroblasts associated with normal breast epithelium, and these alterations in carcinoma-associated fibroblasts (CAF) may promote breast carcinogenesis and cancer progression. A better understanding of the changes that occur in fibroblasts during carcinogenesis and their influence on epithelial cell growth and behavior could lead to novel strategies for the prevention and treatment of breast cancer. To this end, the effect of CAF and normal breast-associated fibroblasts (NAF) on the growth of epithelial cells representative of pre-neoplastic breast disease was assessed.

**Methods:**

NAF and CAF were grown with the nontumorigenic MCF10A epithelial cells and their more transformed, tumorigenic derivative, MCF10AT cells, in direct three-dimensional co-cultures on basement membrane material. The proliferation and apoptosis of MCF10A cells and MCF10AT cells were assessed by 5-bromo-2'-deoxyuridine labeling and TUNEL assay, respectively. Additionally, NAF and CAF were compared for expression of insulin-like growth factor II as a potential mediator of their effects on epithelial cell growth, by ELISA and by quantitative, real-time PCR.

**Results:**

In relatively low numbers, both NAF and CAF suppressed proliferation of MCF10A cells. However, only NAF and not CAF significantly inhibited proliferation of the more transformed MCF10AT cells. The degree of growth inhibition varied among NAF or CAF from different individuals. In greater numbers, NAF and CAF have less inhibitory effect on epithelial cell growth. The rate of epithelial cell apoptosis was not affected by NAF or CAF. Mean insulin-like growth factor II levels were not significantly different in NAF versus CAF and did not correlate with the fibroblast effect on epithelial cell proliferation.

**Conclusion:**

Both NAF and CAF have the ability to inhibit the growth of pre-cancerous breast epithelial cells. NAF have greater inhibitory capacity than CAF, suggesting that the ability of fibroblasts to inhibit epithelial cell proliferation is lost during breast carcinogenesis. Furthermore, as the degree of transformation of the epithelial cells increased they became resistant to the growth-inhibitory effects of CAF. Insulin-like growth factor II could not be implicated as a contributor to this differential effect of NAF and CAF on epithelial cell growth.

## Introduction

The structure and homeostasis of normal breast parenchyma is maintained by dynamic interactions between breast epithelial cells and their associated stroma. These stromal elements include the vasculature, adipocytes, resident immune cells, and fibroblasts with their numerous cellular products, including various growth factors and extracellular matrix (ECM) components. In breast cancers, the stroma differs from stroma found in normal breast. The stromal alterations that accompany most invasive breast carcinomas are morphologically characterized by an enhanced accumulation of fibroblasts and a modified, collagenized extracellular matrix. Breast carcinoma-associated fibroblasts (CAF) have been reported to express increased amounts of specific ECM molecules, various molecules that modulate the ECM, and several peptide growth factors, including insulin-like growth factor (IGF) II, in comparison with fibroblasts in histologically normal breast (i.e. normal breast-associated fibroblasts [NAF]) [1,2].

Fibroblasts surrounding ductal carcinoma *in situ *(DCIS), prior to the development of invasive carcinoma, also differ from those in histologically normal breast tissue. Some of the molecular alterations found in CAF also have been documented, by immunohistochemistry and by *in situ *hybridization, in the fibroblasts surrounding the DCIS [3-6]. This suggests an accumulation of alterations in stromal fibroblasts (i.e. a progression from NAF to CAF) surrounding the breast epithelium as it progresses from normal to hyperplasia to DCIS and invasive cancer.

The role that these stromal changes play in the development and progression of breast cancer and their effect on fibroblast–epithelial cell interactions is a current topic of much interest. It is theorized that CAF act to enhance breast cancer progression [2], and much of the experimental evidence to date supports this contention [7-14]. However, assessment of the effect of CAF has concentrated on established breast cancers. The focus in the present article is on the effect of fibroblasts on the growth of epithelial cells derived from benign breast disease, specifically proliferative breast disease (i.e. MCF10A cells and MCF10AT cells), and the potential role of fibroblast–epithelial cell interactions to promote the development of breast cancer. If CAF promote epithelial cell growth to a greater degree than NAF, could preventing the alterations that occur in fibroblasts surrounding epithelial lesions during carcinogenesis inhibit the progression of the epithelial lesion? To address this possibility, the key signaling and regulatory pathways mediating the effects of fibroblast–epithelial cell interactions, as well as the way in which these interactions are altered during carcinogenesis, must be identified.

Studies to date indicate that the IGF system may play a role in the stromal–epithelial interactions that affect breast cancer progression. IGF I and IGF II both function in cellular growth, in differentiation, and in survival in all tissues. Signaling by IGF I and IGF II through their principal receptor, the insulin-like growth factor receptor, can promote cell cycle progression and can inhibit apoptosis [15]. In breast cancer, the normal regulation and functioning of the IGF system is altered, and the insulin-like growth factor receptor is expressed in 39–93% of breast cancers [16]. By *in situ *mRNA analysis and immunohistochemistry, IGF II expression is reported to be increased in the stromal cells within some breast cancers in comparison with the stromal cells adjacent to normal breast epithelium [17]. In the present study, the effect of NAF and CAF on the growth of pre-cancerous breast epithelial cells was compared. To explore a potential role for IGF II in the growth modulation of breast epithelial cells by NAF and CAF, the level of expression of IGF II in CAF versus that in NAF was assessed.

NAF and CAF were grown in direct contact co-cultures with MCF10A breast epithelial cells and MCF10AT breast epithelial cells, both of which are considered representative of pre-invasive breast disease. MCF10A cells were derived from benign proliferative breast disease. These cells carry a deletion of the chromosomal locus containing p16 and p14ARF, and amplification of MYC [18,19]. MCF10A cells were transfected with mutated T24 H-ras to yield the MCF10AT cells. When suspended in the basement membrane material Matrigel^®^, the MCF10AT cells persist as xenografts in nude mice. The cells initially form structures that resemble normal breast epithelium, and then gradually undergo transition to structures resembling proliferative breast disease and DCIS. Approximately 25% of the MCF10AT xenografts develop invasive carcinoma. The MCF10AT model thus reflects temporally and morphologically high-risk human proliferative breast disease [20,21].

Our *in vitro *model consists of a three-dimensional (3D) direct co-culture system in Matrigel^® ^similar to that utilized by Debnath and colleagues and by Shekhar and colleagues [19,22]. A 3D system was selected over standard monolayer cultures because it more closely simulates *in vivo *growth [19]. When grown in Matrigel^®^, human luminal breast epithelial cells, primary or immortalized, form spherical polarized structures that resemble normal lobular acini. This spatial organization of cells determines how cells perceive and respond to signals from the stromal microenvironment [23].

Importantly, it has been demonstrated that intracellular signaling directing the proliferation of breast epithelial cells differs in cells grown in two dimensions versus those grown in three dimensions [23-25]. Incorporation of NAF and CAF in this 3D culture system allowed assessment of soluble and insoluble secreted factors and of direct contact factors in the fibroblast–epithelial interactions influencing epithelial cell growth.

## Methods

### Maintenance of epithelial cell lines

MCF10A cells (American Type Culture Collection, Manassas, VA, USA) and MCF10AT cells (Karmanos Cancer Institute, Detroit, MI, USA) were cultivated in DMEM/Ham's F-12 (Cambrex, Walkersville, MD, USA) supplemented with 0.1 μg/ml cholera toxin (Calbiochem, San Diego, CA, USA), 10 μg/ml insulin (Sigma, St Louis, MO, USA), 0.5 μg/ml hydrocortisone (Sigma), 0.02 μg/ml epidermal growth factor (Upstate Biotechnology, Lake Placid, NY, USA) and 5% horse serum (Invitrogen, Carlsbad, CA, USA). Subconfluent cultures (80–90% confluence) were utilized in experiments.

### Isolation, characterization and maintenance of fibroblast cultures

Fibroblasts were derived from mammary reduction specimens (NAF) and from primary breast cancers (CAF). The tissues were remnants of diagnostic surgical specimens and were obtained from The University of Alabama at Birmingham Tissue Procurement Facility after Institutional Review Board approval. H&E-stained, frozen histologic sections were prepared from each tissue sample to confirm benignity or malignancy. The tissue samples from the breast reduction specimens consisted predominantly of adipose tissue, but interspersed fibrous areas were selected for fibroblast isolation. The tissue was minced and digested for 18–24 hours at 37°C in DMEM (Vitacell, Manassas, VA, USA) with 10% fetal bovine serum (Invitrogen) supplemented with 100 U/ml streptomycin, 100 μg/ml penicillin, 2.5 μg/ml Fungizone (GibcoBRL, Life Technologies, Grand Island, NY, USA), 150 U/ml hyaluronidase (Sigma) and 200 U/ml collagenase type III (GibcoBRL). The digested tissue was centrifuged at 100 relative centrifugal force and plated in T25 tissue culture flasks with DMEM and 10% fetal bovine serum. Differential trypsinization was applied during subculturing to select for the growth of fibroblasts [26].

Early passages (below passage 9) of all fibroblasts were subjected to immunocytochemical evaluation with anti-vimentin (mouse IgG_1_, clone V9; Neomarkers, Fremont, CA, USA), anti-epithelial membrane antigen (mouse IgG_2a_, clone ZCE113; Zymed, San Francisco, CA, USA), and anti-cytokeratin (CK) 5/CK 8 (mouse IgG_1_, clone C-50; Neomarkers) as confirmation of their stromal origin (i.e. strong vimentin expression, and absence of epithelial membrane antigen and CK 5/CK 8). Epithelial membrane antigen and CK 8 are expressed primarily in luminal breast epithelial cells, whereas CK 5 is found in myoepithelial cells [27]. For immunocytochemical evaluation, fibroblasts were grown on glass coverslips, fixed in 70% ethanol and were permeabilized with acetone. Fibroblasts were incubated in 3% hydrogen peroxide followed by 3% goat serum at room temperature. Anti-vimentin (0.5 μg/ml), anti-epithelial membrane antigen (1 μg/ml), or anti-CK 5/CK 8 (0.3 μg/ml) were applied for 1 hour at room temperature. Secondary detection was accomplished with a streptavidin/horseradish peroxidase secondary detection system (Signet Laboratories, Dedham, MA, USA) and diaminobenzidine (BioGenex, San Ramon, CA, USA). Harris hematoxylin was used as a counterstain.

The fibroblasts were routinely maintained in DMEM and 10% fetal bovine serum. Subconfluent cultures (70–90% confluence) of lower passages (below passage 9) were utilized for the experiments described. Only early-passage fibroblast cultures are used in experiments to more closely simulate their *in vivo *phenotype. It has been shown that early-passage (below passage 9) colonic primary fibroblasts maintain many of their *in vivo *characteristics, including expression of alpha-smooth muscle actin, collagen IV and laminin 1 [28]. Multiple NAF and CAF cultures were utilized because of potential variation among fibroblasts from different individuals and different breast cancers.

### Preparation of the 3D cultures

The effect of NAF and CAF on MCF10A breast epithelial cells and MCF10AT breast epithelial cells was studied using a 3D *in vitro *model. In co-cultures, epithelial cells and fibroblasts were mixed in Human Endothelial–SFM Basal Growth Media (Invitrogen) supplemented with 10 ng/ml epidermal growth factor and 20 ng/ml basic fibroblast growth factor (Invitrogen). This was followed by dispersal on 100 μl basement membrane material (Growth Factor Reduced Matrigel^®^; BD Biosciences, Bedford, MA, USA) in each well of eight-well chamber slides (Lab-Tek^® ^Chamber Slide™ System; Nalge Nunc International, Naperville, IL, USA).

The ratio of epithelial cells to fibroblasts (E:F) ranged from 2:1 to 1:3 by varying the number of fibroblasts while keeping the number of epithelial cells constant (100,000 cells/well). Controls consisted of 3D monocultures of MCF10A cells and MCF10AT cells (100,000 cells/well). All cultures were incubated in a 37°C, 5% CO_2 _humidified incubator for 14 days with supplementation of fresh Human Endothelial-SFM Basal Growth Media supplemented with 10 ng/ml epidermal growth factor and 20 ng/ml basic fibroblast growth factor at 4-day intervals.

Morphologic development was observed by phase contrast microscopy. 5-bromo-2'-deoxyuridine (BrdU) (0.2 mg/ml; Calbiochem) was applied to all cultures for 24 hours. The cultures were removed from the chamber slides, were embedded in HistoGel specimen processing gel (Richard-Allan, Kalamazoo, MI, USA), were fixed in 10% neutral-buffered formalin, and were embedded in paraffin. Histologic sections were prepared for H&E staining and immunocytochemistry.

### BrdU detection by immunocytochemistry

Distinction of epithelial cells from fibroblasts in the 3D cultures was accomplished by examination of the cell morphology and location within the culture. To ensure that only epithelial cells were counted, however, immunostaining with anti-BrdU (mouse IgG_1_, clone Bu20a; DAKO, Carpinteria, CA, USA) was followed by immunostaining with anti-CK 5/CK 8, expressed only in MCF10A cells and MCF10AT cells in 3D cultures. The staining entailed pretreatment with low-temperature antigen retrieval (i.e. incubation in 0.01 M citric acid monohydrate, pH 6.0, for 2 hours in an 80°C water bath), followed by sequential incubation in 1 N HCl, 3% hydrogen peroxide, 1% goat serum, and anti-BrdU (3 μg/ml). Secondary detection was as previously described. This was followed by incubation with anti-CK 5/CK 8 (4 μg/ml) and secondary detection using a streptavidin/alkaline phosphatase reagent (Signet Laboratories) and New Fuchsin (BioGenex) as the chromogen. The slides were lightly counterstained with Harris hematoxylin.

The resulting dual coloration of anti-BrdU (brown nucleus) and anti-CK 5/CK 8 (pink cytoplasm) enabled identification of proliferating epithelial cells. A BrdU-labeling index in the epithelial cells was determined by calculating the percentage of epithelial cells with nuclear staining for anti-BrdU in complete cross-sections of the 3D cultures. A minimum of 500 epithelial cells was counted. Negative controls consisted of histologic sections of each 3D culture processed without the addition of primary antibodies.

### BrdU detection by flow cytometry

Cells were gently removed from 3D cultures after treatment with Dispase (BD Biosciences Discovery Labware, Bedford, MA, USA), followed by 5–10 mM EDTA. Recovered cells were washed with cold PBS. To distinguish co-cultured epithelial cells from fibroblasts, allophycocyanin-conjugated anti-EpCAM (mouse IgG_1_, clone EBA-1; BD Biosciences Immunocytometry Systems, San Jose, CA, USA) was used to label MCF10A cells and MCF10AT cells. The cells were permeabilized and fixed (BD Cytofix/Cytoperm and Perm/Wash kit; BD Biosciences Pharmingen, San Diego, CA, USA). Prior to staining with FITC-conjugated anti-BrdU (mouse IgG_1_, clone B44; BD Biosciences Immunocytometry Systems), cells were treated with DNase I (Roche Diagnostics GmbH, Penzberg, Germany). Samples were analyzed on a BD FACS Calibur™ flow cytometer (BD Biosciences). The percentage of BrdU-labeled epithelial cells (positive for anti-EpCAM and anti-BrdU) was calculated from the total acquired events.

### Assessment of apoptosis by TUNEL assay

Apoptosis was quantified in epithelial cells in 3D cultures by the TUNEL Assay (ApopTag Peroxidase* In Situ* Apoptosis Kit; Integren Co., Purchase, NY, USA) as per the manufacturer's instructions. In the TUNEL assay, terminal deoxynucleotide transferase was used to label fragmented DNA with digoxigenin-linked nucleotides. These nucleotides were then detected using an anti-digoxigenin antibody. Negative controls consisted of histologic sections of each 3D culture processed without the addition of terminal deoxynucleotide transferase. Sequential sections of each 3D culture were stained with anti-CK 5/CK 8, as previously described, and were used to aid in identification of epithelial cells in co-cultures. The percentage of epithelial cells with nuclear staining was determined in complete cross-sections of the 3D cultures. A minimum of 500 epithelial cells was counted.

### Preparation of cell lysates

Cells were gently removed from Matrigel^® ^in 3D co-cultures of MCF10AT cells (100,000 cells/well) with fibroblasts (50,000 cells/well) and monocultures of MCF10AT cells (100,000 cells/well) or fibroblasts (50,000 cells/well) using dispase, as previously described. The released cells were washed with cold PBS to remove residual Matrigel^®^. Cell lysates were prepared from 3D cultures and monolayer cultures with NEB lysis buffer (Cell Signaling Technology, Beverly, MA, USA) containing 20 mM Tris–HCl (pH 7.5), 150 mM NaCl, 1 mM Na_2_EDTA, 1 mM EGTA, 1% Triton, 2.5 mM sodium pyrophosphate, 1 mM β-glycerophosphate, 1 mM Na_3_VO_4_, 1 μg/ml leupeptin, 10 mM sodium fluoride, a Complete Mini Protease Inhibitor Cocktail tablet (Roche, Indianapolis, IN, USA) and 1 mM phenylmethylsulfonylfluoride, and were cleared by centrifugation. All lysates were concentrated with Millipore Microcon^® ^Centrifugal Filter Devices YM-3 (Millipore Corporation, Bedford, MA, USA). The protein concentration of lysates was measured with the BCA Protein Assay Kit (Pierce, Rockford, IL, USA).

### Enzyme-linked immunosorbent assay

The protein levels of IGF II in cell lysates were measured by ELISA for human IGF II (DSL-10-9100 Active™ IGF-II ELISA; Diagnostic System Laboratories, Webster, TX, USA) as per the manufacturer's instructions. The kit includes a modified version of the standard acid–ethanol extraction prior to ELISA. The ELISA for IGF II shows no detectable cross-reactivity with IGF I, insulin, IGF binding protein 1, and IGF binding protein 3. The minimum detection limit of the assay is 0.25 ng/ml. The amount of IGF II present in lysates from cell lines and 3D cultures was normalized to the total protein concentration.

### Quantitative real-time PCR

RNA isolation (Trizol reagent; GibcoBRL) was followed by RNA clean-up with RNeasy columns (Qiagen, Valencia, CA, USA). Spectrophotometric ratios of A_260 _to A_280 _were greater than 1.8. The forward, reverse and probe oligonucleotides were synthesized and purified by HPLC (Applied Biosystems, Foster City, CA, USA) after complete evaluation of the IGF II and ribosomal S9 gene sequences (GenBank database) (IGF II forward, 5'-GTCGATGCTGGTGCTTCTCA-3' and reverse, 5'-GGGCGGTAAGCAGCAATG-3' ; probe, 5'-6FAMCTTCTTGGCCTTCGCCTCGTGCTTAMRA-3' ; ribosomal S9 forward, 5'-ATCCGCCAGCGCCATA-3' and reverse, 5'-TCAATGTGCTTCTGGGAATCC-3' ; probe, 5'-6FAMAGCAGGTGGTGAACATCCCGTCCTTTAMRA-3') using the Primer Express software (Applied Biosystems).

Fluorescent signal data were collected by the ABI Prism 7700 Sequence Detection System (Applied Biosystems). The log-linear phase of amplification was monitored to obtain the threshold cycle (defined as the fractional cycle number at which the amount of the amplified target reaches a fixed threshold) values for each RNA sample. Ribosomal S9 was used as the internal reference and was selected because it exhibits minimal variability in tissues of different origins [29]. The comparative threshold cycle method was employed to determine IGF II expression levels in each sample relative to a calibrator, in this case MCF10AT cells [29,30]. Each sample was run in triplicate.

### Statistical analysis

A relationship between BrdU labeling or apoptotic indexes and co-culture with NAF or with CAF was assessed by multiple linear regression analysis to allow combining replicate experiments performed on different days. Results of ELISA and quantitative real-time PCR were compared by *t *test. Outliers were eliminated prior to analysis using a basic outlier test, where a high outlier was defined as a number greater than quartile 3 + 1.5 (interquartile range) and a low outlier was less than quartile 1 – 1.5 (interquartile range).

## Results

### Fibroblasts alter the 3D morphology of MCF10A cells and MCF10AT cells

The immunocytochemical characterization of fibroblasts used in the described experiments confirmed their stromal nature. Immunostaining for vimentin was strongly positive and staining for epithelial membrane antigen and CK 5/CK 8 was negative.

**Figure 1:**
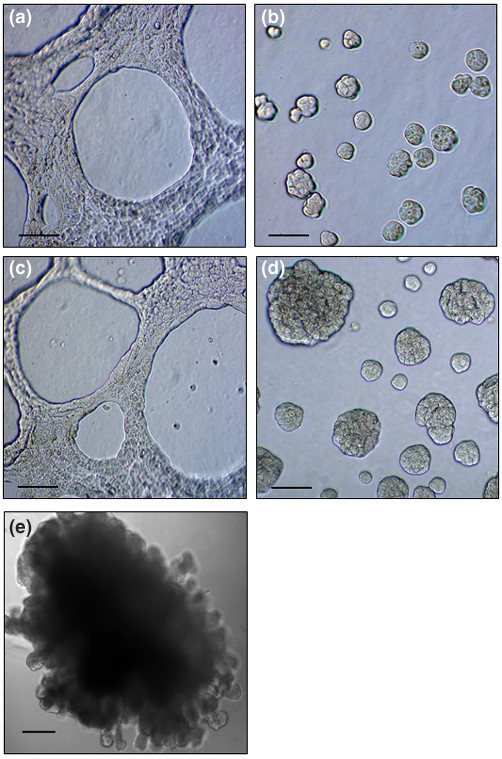
MCF10A cells and MCF10AT cells in monoculture and in co-culture with fibroblasts. **(a)**, **(b) **MCF10A cells and **(c)**, **(d) **MCF10AT cells in monoculture initially form a lattice/scaffold arrangement (a, c). After several days of culture, spheroidal structures become more prominent (b, d). **(e) **MCF10AT cells in co-culture with fibroblasts form three-dimensional rounded structures. Similar structures are formed by MCF10A cells in co-culture with fibroblasts (phase contrast, 100 × magnification; scale bar, 200 μm).

**Figure 2:**
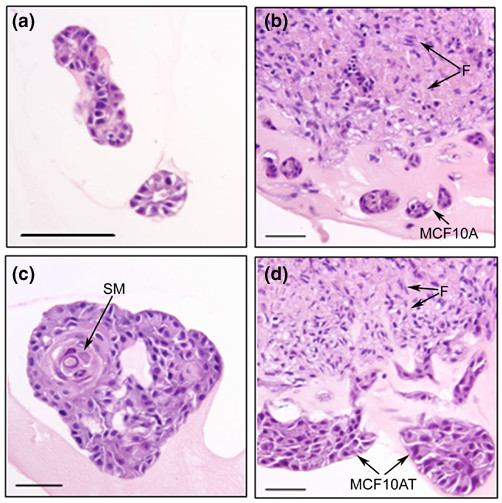
H&E-stained histologic sections of MCF10A cell and MCF10AT cell monocultures and co-cultures with fibroblasts. **(a) **MCF10A cells form small spheroids. **(b) **MCF10A cells in co-culture with fibroblasts are located adjacent to the fibroblast aggregate (F) and maintain smaller spheroids. **(c) **In monoculture, MCF10AT cells form larger rounded three-dimensional structures. **(d) **In co-culture, MCF10AT cells form solid sheets and rounded groups of cells located adjacent to the fibroblasts (F). The occurrence of squamous metaplasia (SM) is more evident in MCF10AT monocultures, while it is suppressed in co-cultures. Overall, MCF10A cells have less squamous metaplasia than MCF10AT cultures (400 × magnification; scale bar, 50 μm).

In 3D monocultures, both MCF10A cells and MCF10AT cells initially form a lattice-like network of duct-like structures. After several days, the lattice-like network is replaced by a predominance of rounded epithelial cell groups (spheroids) (Fig. 1a,1b,1c,1d). MCF10A cells form small, rounded spheroids (Figs 1b and 2a). MCF10AT cells aggregate into larger solid groups or sheets with extensive squamous metaplasia (Figs 1d and 2c). The formation of larger three-dimensional structures and the abnormal differentiation (i.e. squamous metaplasia) of MCF10AT cells supports their greater degree of transformation.

**Figure 3:**
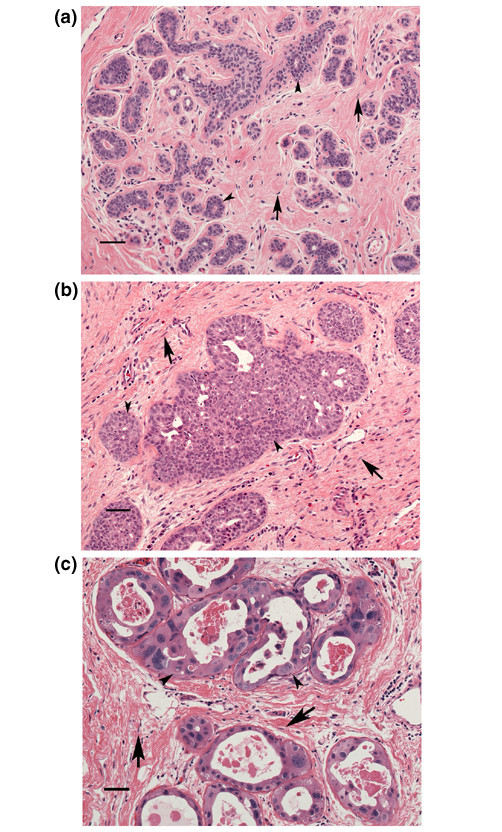
Distribution and relative quantities of fibroblasts and epithelial cells in a normal terminal duct-lobular unit, hyperplasia and ductal carcinoma *in situ *(DCIS). **(a) **A terminal duct-lobular unit with epithelial cells (arrowheads) arranged in acini and intralobular terminal ducts separated by stroma-containing fibroblasts (arrows) in a ratio of epithelial cells to fibroblasts (E:F) of 2.7:1. **(b) **Ductal hyperplasia with a proliferation of epithelial cells (arrowheads) filling and expanding terminal ducts separated by reactive stroma including fibroblasts (arrows) in an E:F of 3.3:1. **(c) **High-grade DCIS, with epithelial cells (arrowheads) demonstrating markedly atypical nuclei, involving terminal ducts separated by reactive stroma including fibroblasts (arrows) in an E:F of 2:1 (200 × magnification; scale bar, 50 μm).

In 3D co-cultures with NAF or with CAF, the epithelial cells and fibroblasts form large rounded structures (Fig. 1e). In histologic sections of co-cultures with NAF or with CAF, MCF10A cells and MCF10AT cells form spheroids or sheets within Matrigel^®^, as seen in monocultures, and these epithelial groups surround an aggregate of fibroblasts (Fig. 2b,2d). These 3D co-cultures resemble a terminal duct-lobular unit in the normal breast or in proliferative breast disease/DCIS (Fig. 3a,3b,3c), in that epithelial cells are arranged in groups (similar to an acinus or a terminal duct) surrounded by a laminin-rich basement membrane with fibroblasts located outside the basement membrane and separating epithelial groups. The 3D *in vitro *model differs from normal breast or *in situ *breast disease *in vivo*, however, in that the number of fibroblasts centrally located and between epithelial cell groups is greater than that typically seen *in vivo*. Additionally, the fibroblasts in the *in vitro *model are surrounded by an ECM rich in laminin and collagen IV [31], whereas *in vivo *collagen I would typically predominate [32].

In 3D co-cultures, both NAF and CAF markedly suppressed squamous metaplasia of MCF10AT cells as observed on H&E-stained sections of monocultures, thereby normalizing the morphology to a glandular phenotype (Fig. 2c,2d). No obvious differences between NAF and CAF in morphology, growth pattern or adhesion to substrate, in either a monolayer or the 3D culture, were observed.

### NAF and CAF affect the rate of proliferation of MCF10A cells and MCF10AT cells in 3D co-culture

**Figure 4:**
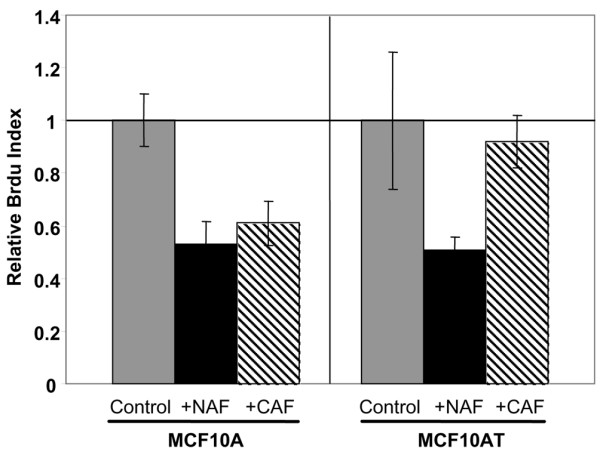
Proliferation of MCF10A cells and MCF10AT cells grown in monoculture and co-culture with fibroblasts. The rate of proliferation of MCF10A cells and MCF10AT cells, as measured by the 5-bromo-2'-deoxyuridine (BrdU) labeling index (assessed by immunocytochemistry), was significantly reduced in co-cultures of MCF10A cells with both normal breast-associated fibroblasts (NAF) (*P *= 0.009) and carcinoma-associated fibroblasts (CAF) (*P *= 0.024) compared with the MCF10A monoculture (control). The rate of proliferation of MCF10AT cells was significantly suppressed by NAF (*P *= 0.013) but not by CAF (*P *= 0. 935) in comparison with the MCF10AT monoculture (control).

**Figure 5:**
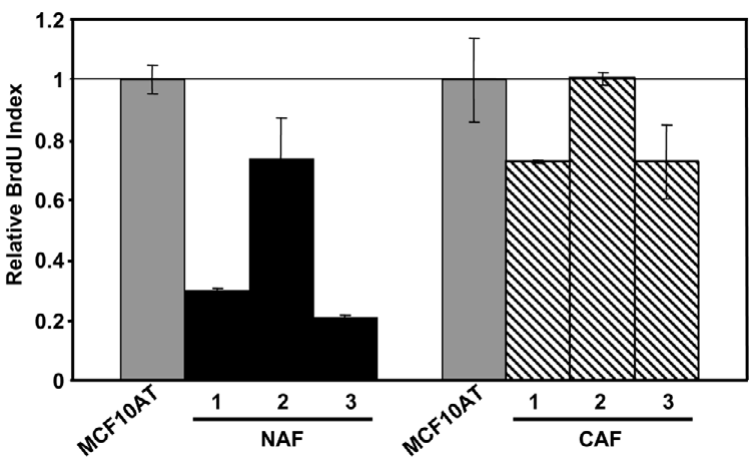
5-Bromo-2'-deoxyuridine (BrdU) labeling, assessed by flow cytometry, of MCF10AT monocultures and co-cultures with normal breast-associated fibroblasts (NAF) and carcinoma-associated fibroblasts (CAF). These data are representative of replicate experiments indicating that NAF suppress proliferation of MCF10AT cells to a greater extent than do CAF. Again some variability in extent of suppression is present among individual NAF cultures and individual CAF cultures.

In replicate co-cultures of MCF10A cells with three different NAF and CAF grown in an E:F of 2:1, both types of fibroblasts significantly reduced proliferation of MCF10A cells. The mean BrdU-labeling index of MCF10A cells, when measured by immunocytochemistry, was decreased by 47% in co-culture with NAF (*n *= 19, *P *= 0.009) and by 39% in co-culture with CAF (*n *= 19, *P *= 0.024) relative to the MCF10A monoculture (Table 1 and Fig. 4). The BrdU-labeling index of MCF10AT cells was reduced by 49% in the presence of NAF (*n *= 20, *P *= 0.013), relative to the MCF10AT monoculture, whereas co-culture with CAF failed to significantly lower the MCF10AT BrdU-labeling index (*n *= 22, *P *= 0.935) (Table 2 and Fig. 4). The effect of NAF versus CAF on the rate of proliferation of MCF10AT cells was significantly different (*P *< 0.001). The effect was further confirmed by repeating the co-cultures to measure the BrdU-labeling index by flow cytometry, rather than by immunocytochemistry (Fig. 5).

There was variability among NAF cultures and among CAF cultures in their ability to suppress proliferation of MCF10A cells and MCF10AT cells (Tables 1 and 2) in this 3D culture system, potentially reflecting heterogeneity among the individuals from which the fibroblasts were derived. Because of this variability, detection of a significant difference in the function of NAF and CAF required many replicates and multiple fibroblast cultures derived from different individuals.

**Figure 6:**
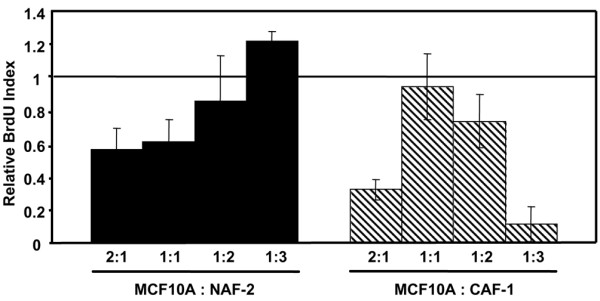
Relative 5-bromo-2'-deoxyuridine (BrdU) indices of MCF10A cells in co-culture with varying quantities of normal breast-associated fibroblast NAF-2 and carcinoma-associated fibroblast CAF-1. With increasing numbers of NAF-2, the mean rate of proliferation of co-cultured MCF10A cells increased, with a significant difference in BrdU-labeling index observed between a ratio of epithelial cells to fibroblasts (E:F) of 2:1 versus an E:F of 1:3 (*P *< 0.05). With increasing numbers of CAF-1, the mean rate of proliferation was highest at an E:F of 1:1. The rate of proliferation at an E:F of 1:1 was significantly higher than that at an E:F of 2:1 (*P *< 0.05). At an E:F of 1:3, CAF-1 caused a decreased proliferation of and enhanced cell death of MCF10A cells.

In a prior report, CAF was found to promote, rather than inhibit, the growth of MCF10A cells in a similar 3D co-culture system [22]. One of several possible explanations for this discrepancy between the prior result and the present result is a difference in E:F. Shekhar and colleagues used an E:F of 1:1 rather than the E:F of 2:1 we initially used [22]. The number of fibroblasts has been shown to have an effect on the response of epithelial cells [7,9,14]. We therefore repeated the 3D co-cultures of MCF10A cells using NAF-2 and CAF-1 with increasing numbers of fibroblasts (i.e. a decreasing E:F) (Fig. 6). BrdU labeling was assessed by immunocytochemistry of histologic sections of 3D cultures.

As previously, NAF-2 at an E:F of 2:1 suppressed proliferation of MCF10A cells. However, with increasing numbers of NAF-2, this suppression effect was gradually weakened (*P *= 0.043). Although we found no significant difference in the suppressive effect of NAF-2 in an E:F of 2:1 versus an E:F of 1:1 or of 1:2, there was a significantly greater rate of proliferation of MCF10A cells with NAF-2 in an E:F of 1:3 compared with in an E:F of 2:1 (*P *= 0.028). More importantly, CAF-1 at an E:F of 1:1 did not significantly suppress proliferation, whereas our original ratio of 2:1 did (*P *= 0.025). CAF-1 at an E:F of 1:2 also conferred a higher rate of proliferation of MCF10A cells than the E:F of 2:1, but this did not reach statistical significance (*P *= 0.054). At an E:F of 1:3, however, CAF-1 caused a decrease in proliferation of MCF10A cells and enhanced cell death, as assessed by microscopic morphology. At an E:F of 1:3, the total number of viable MCF10A cells was reduced in co-culture with both NAF-2 and CAF-1; however, this reduction was more marked with CAF-1.

**Figure 7:**
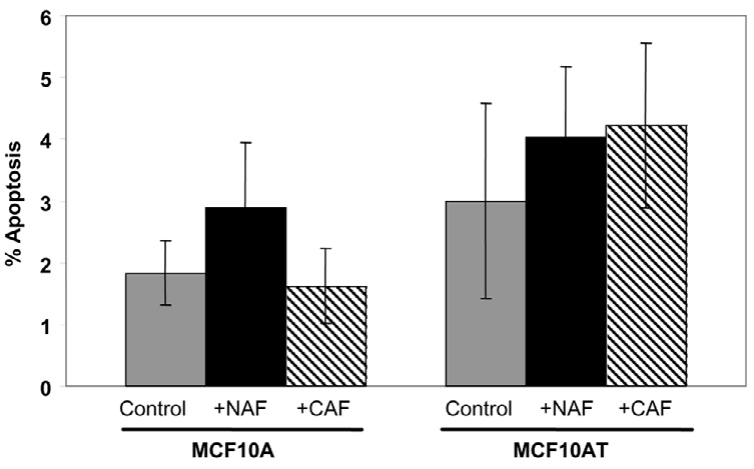
TUNEL assay for MCF10A cells and MCF10AT cells in co-culture with normal breast-associated fibroblasts (NAF) and carcinoma-associated fibroblasts (CAF). Co-culture with NAF and CAF had no significant effect on the rate of apoptosis of MCF10A cells and MCF10AT cells in comparison with epithelial cell monoculture controls.

Neither NAF nor CAF had a significant effect on the rate of apoptosis of MCF10A cells or MCF10AT cells when grown at an E:F of 2:1 after 2 weeks of co-culture, as assessed by TUNEL assay (Fig. 7).

### Quantities of IGF II are no different in NAF versus CAF

As an initial attempt to identify differences between NAF and CAF that explain our observed results, expression of IGF II in NAF and in CAF was assessed. A higher level of expression of IGF II in CAF than in NAF may provide an explanation for the higher rate of proliferation of MCF10AT cells allowed by CAF in comparison with NAF.

ELISA performed on cell lysates of NAF and CAF cultures demonstrated variability in expression of IGF II among cultures, but no significant difference was observed in the mean IGF II quantity between NAF and CAF in monolayer cultures (Table 3) or in 3D monocultures (Table 4). Although in monolayer cultures more CAF than NAF had IGF II levels > 10 ng/μg protein, the mean levels of IGF II were similar (NAF versus CAF, *P *> 0.05) (Table 3). This comparison included five different NAF cultures and six different CAF cultures. ELISA was also performed on NAF-conditioned media and CAF-conditioned media but, despite the concentration of samples, the levels were too low for reliable quantification by ELISA (data not shown).

**Figure 8:**
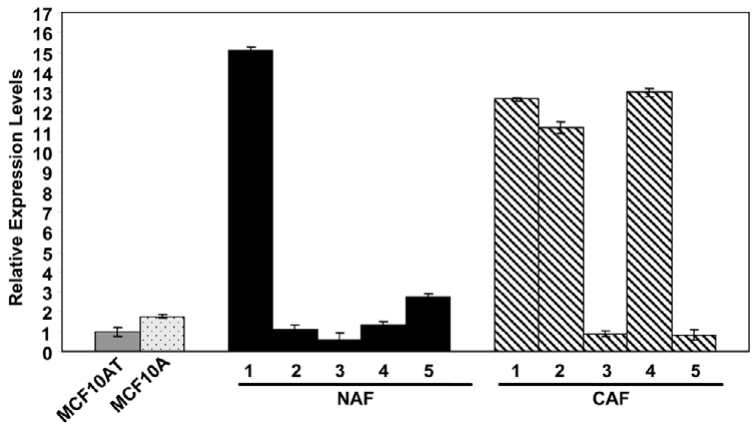
Comparative expression of insulin-like growth factor (IGF) II mRNA. IGF II expression levels in MCF10A cells, MCF10AT cells, normal breast-associated fibroblasts (NAF) and carcinoma-associated fibroblasts (CAF) were determined by quantitative real-time PCR. All expression levels are relative to the calibrator, MCF10AT cells. The error bars represent the standard deviation of triplicate assays for each sample. Comparison of the mean expression level between NAF (mean = 4.2) and CAF (mean = 7.7) did not reach statistical significance (*P *= 0.39, *t *test).

Additionally, because IGF II mRNA was previously reported to be expressed at a higher level in CAF than in NAF, IGF II mRNA was assessed by quantitative real-time PCR in monolayer cultures of NAF and CAF. The relative expression levels for each fibroblast culture are provided in Fig. 8. Although more CAF cultures than NAF cultures expressed IGF II mRNA at relatively high levels, the mean relative expression level of IGF II mRNA for NAF (4.2, *n *= 5) and for CAF (7.7, *n *= 5) did not differ significantly (*P *= 0.390).

Co-culture of MCF10A cells or MCF10AT cells may enhance expression of IGF II in fibroblasts, as has been reported in fibroblasts co-cultured with MCF-7 cells [33]. Furthermore, expression of IGF II may be enhanced to a greater degree in CAF versus NAF, thus possibly explaining the greater rate of proliferation of MCF10AT cells in co-culture with CAF than with NAF. 3D culture of NAF and CAF also could alter expression of IGF II in comparison of the monolayer culture. To investigate these possibilities, IGF II levels were assessed by ELISA in 3D monocultures of the same three NAF cultures and three CAF cultures used in previous co-cultures, in 3D monocultures of MCF10AT cells, and in 3D co-cultures of MCF10AT cells with NAF and CAF in an E:F of 2:1, identical to those co-cultures assessed for BrdU labeling previously (Table 4). In the 3D cultures, fibroblasts expressed IGF II at a significantly higher level than the epithelial cells (*P *< 0.01), and there was no difference in IGF II between NAF and CAF (*P *> 0.05). In co-cultures, the overall expression of IGF II was lower than in fibroblast monocultures. This latter result is expected because of the addition of a relatively large number of MCF10AT cells (E:F of 2:1) that express IGF II at a lower level than fibroblasts. Furthermore, there was no difference in mean IGF II levels in co-cultures with NAF versus CAF. The IGF II levels in 3D cultures were generally higher than in monolayer cultures, and this was particularly true when comparing 3D monocultures of fibroblasts versus two-dimensional monocultures of fibroblasts. The potential reasons for this include an effect of 3D growth, or the presence of Growth Factor Reduced Matrigel^® ^on the expression of IGF II, and/or deviations between the ELISA runs.

When the protein levels of IGF II in 3D monocultures of fibroblasts or in 3D co-cultures were correlated with the rate of proliferation of MCF10AT cells in co-culture with the matching NAF or CAF, no significant correlation was observed (*r *= 0.030 or *r *= 0.258, respectively; *P *> 0.05, Pearson Product Moment Correlation).

## Discussion

Our results indicate that both NAF and CAF have the ability to inhibit epithelial cell proliferation and to induce glandular differentiation to a more normal phenotype. However, CAF have less inhibitory capacity than NAF. At relatively low concentrations of fibroblasts (E:F of 2:1) NAF can suppress proliferation of both MCF10A cells and MCF10AT cells, whereas CAF can suppress proliferation of MCF10A cells but not the more transformed MCF10AT cells. *In vivo *NAF may thus have an inhibitory and regulatory effect on the proliferation of normal epithelial cells. This suppressive ability may be lost or reduced as epithelial lesions gradually progress from hyperplasia to DCIS and invasive cancer, and correspondingly NAF become CAF.

Differences in gene expression between CAF and NAF have been documented by examination of human breast cancers by immunohistochemistry and *in situ *hybridization, and also by analysis of cultures of fibroblasts isolated from breast cancers. These documented characteristics of breast-derived CAF are thoroughly reviewed by Kunz-Schughart and Knuechel [1,2], and include an increased expression of several growth factors [3,34], of ECM molecules [35-37], and of proteases and protease inhibitors involved in modulating the ECM [5,6]. Many of these differences in the expression profile of CAF in comparison with NAF have the potential to enhance the development, growth and progression of breast carcinoma [1]. Furthermore, a subset of the phenotypic alterations documented in CAF have been identified in fibroblasts surrounding DCIS by examining these lesions using immunohistochemistry or *in situ *hybridization [3-6]. However, many of the changes observed in CAF have not yet been examined in DCIS, and it is therefore quite possible that fibroblasts surrounding DCIS share more features with CAF than are currently documented.

Attempts to actually demonstrate a promotional effect of CAF on the growth of epithelial cell lines derived from breast cancer are limited in number and have met with somewhat conflicting results. A variety of different methods were used in these studies, complicating comparison among them. In most *in vitro *analyses, however, direct and indirect co-culture with CAF increased the growth of MCF-7 breast cancer cells compared with MCF-7 cells alone [7,12,14,38]. Co-culture of NAF with MCF-7 cells caused both growth promotion [7,10,11,13,39,40] and inhibition [8] of MCF-7 cells. In contrast, co-culture of NAF and CAF with the breast cancer cell line MDA-MB-231 had no significant effect on epithelial cell growth [10,11]. *In vivo*, NAF and CAF increased growth of the MCF-7 xenografts or NAF had no effect [7,38]. Only a few reports have addressed the effects of fibroblast–epithelial interactions on the growth of nontransformed or nontumorigenic breast epithelial cells, rather than on the growth of breast carcinoma cells. NAF and CAF have been reported to stimulate the proliferation of normal human breast epithelial cells [7,39] or to have no effect on the rate of proliferation of normal breast epithelial cells immortalized by SV40 large-T antigen [8]. The overriding observations from these previous studies of fibroblast–epithelial interactions are that co-cultured fibroblasts affect the growth of epithelial cells, but this growth is dependent on the source of the fibroblasts, the characteristics of the epithelial cells, and the culture conditions utilized.

In a co-culture system similar to that presented here, NAF (two different cultures) inhibited the growth, measured by direct counting of total viable cells in co-cultures, of MCF10A cells and MCF10AT-EIII8 cells – whereas CAF induced the growth of both epithelial cell lines [22]. While our findings for NAF are similar to the previous results, we did not find a promotional effect of CAF on epithelial cell growth. This discrepancy may be a result of the interindividual variation found in fibroblast cultures and/or differences in epithelial cells (MCF10AT cells versus MCF10AT-EIII8 cells, an estrogen-induced derivative of the MCF10AT cells). In addition, the total number of viable cells present (fibroblasts and epithelial cells) in co-cultures were counted, whereas in the current study only proliferation of epithelial cells was measured. In the previous study, an E:F ratio of 1:1 was utilized compared with the current E:F of 2:1. In support of the latter, we found that CAF at lower E:F values of 1:1 and 1:2 no longer inhibited the growth of MCF10A cells. Although not identical, this result is more in keeping with that of Shekhar and colleagues [22].

Varying E:F ratios have been utilized in a few prior studies of the effect of fibroblasts on the growth of breast carcinoma cells. Ratios have varied from a great predominance of epithelial cells [9] to a predominance of fibroblasts [7,14]. In these previous studies, an increasing proportion of breast fibroblasts, either NAF or CAF, correlates with an increase in growth of co-cultured cancerous breast epithelial cells to a plateau where no further enhancement of growth is seen. This is in general concordance with our results, where the inhibitory effect of both NAF and CAF on proliferation of MCF10A cells was less with increasing numbers of fibroblasts, particularly for NAF. This suggests the presence of fibroblast-derived factors that both inhibit and promote the proliferation of epithelial cells; at higher concentrations of fibroblasts, the promotional effect predominates.

To be biologically relevant, the most meaningful ratio of epithelial cells to fibroblasts depends on the lesion or tissue being modeled. In the present study, the intent was to model proliferative breast disease and DCIS, the putative precursors of invasive carcinoma, which are believed to have their origins in terminal ducts within terminal duct-lobular units [41]. Microscopic examination of such intraductal lesions in the human breast reveals a range of E:F, depending on the extent of fibrosis of the lesion. In the normal terminal duct-lobular unit and *in situ *lesions depicted in Fig. 3, the E:F varies from 3:1 to 2:1. These ratios were determined by counting epithelial cells (both luminal and myoepithelial cells) and stromal cells identified as fibroblasts and located within or in close proximity to the terminal duct-lobular unit involved. It is probable that those fibroblasts in proximity to the epithelial structures have the greatest influence on epithelial cell behavior. Our choice to use an initial E:F of 2:1 is therefore appropriate, whereas lower ratios with many more fibroblasts are less common within terminal duct-lobular units *in vivo*.

Prior attempts to identify fibroblast-derived factors that are mediating the effect of NAF and CAF on breast epithelial cell growth have identified IGF I and/or IGF II as partially contributing to the mitogenic effect of fibroblasts [11]. Previous studies have reported that IGF II was expressed at a moderate to high level in 43–57% of breast cancers by *in situ *hybridization and by immunohistochemistry [17,42,43], with localization primarily to stromal fibroblasts or vessel walls [17,42], making IGF II a potential candidate to mediate fibroblast–epithelial interactions. Additionally, in cultures of CAF and NAF, IGF II mRNA was detected at higher levels more frequently in CAF than in NAF in some studies [33,44], but not in other studies [45,46].

In the current study, IGF II levels in fibroblasts were assessed in both monolayer cultures and 3D cultures by ELISA and quantitative real-time PCR. While more CAF cultures than NAF cultures had relatively higher levels of IGF II mRNA or protein, no significant differences in mean quantities of IGF II mRNA or protein between these CAF and NAF were observed. We also found no difference in IGF II expression in the 3D co-cultures of MCF10AT cells prepared with NAF versus CAF, suggesting that MCF10AT cells do not alter IGF II expression to a different degree in co-cultured NAF and CAF. Furthermore, we found no correlation between proliferation of MCF10AT cells in co-culture and the level of IGF II protein in 3D fibroblast monocultures or 3D co-cultures. Differences in IGF II expression in NAF and CAF are therefore unlikely to explain the difference in the effect of CAF versus that of NAF on proliferation of the epithelial cells described in this study. Our results do not eliminate a role for the IGF system in these fibroblast–epithelial cell interactions as other family members, such as IGF II receptor and a multitude of IGF binding proteins, may be mediating these interactions in other ways.

Prior studies by other workers [33,44-46] and the current work underscore the variability in expression of IGF II and in the growth inhibitory effect of NAF and CAF, and emphasize the importance of including several NAF and CAF cultures from different individuals in studies of fibroblast–epithelial cell interactions. The interindividual heterogeneity observed among NAF and CAF complicates assessment of mechanisms underlying fibroblast–epithelial interactions. Broad generalizations based on the results of experiments using only one or two fibroblast cultures should be avoided.

In conclusion, both NAF and CAF have the ability to suppress breast epithelial cell proliferation; however, the capacity of CAF to inhibit proliferation is less than that of NAF. This suggests that there are differences in either secreted factors or intercellular interactions between NAF and CAF that render CAF less effective in inhibiting proliferation, particularly of more transformed epithelial cells. Furthermore, differences between the phenotypes of the H-ras overexpressing MCF10AT cells and the parental MCF10A cells cause MCF10AT cells to be more resistant to the suppressive effect of fibroblasts. Future work to identify the key fibroblast–epithelial interactions mediating these effects may reveal mechanisms to allow restoration of the inhibitory of effect of fibroblasts during the carcinogenic process.

## Abbreviations

3D = three-dimensional; BrdU = 5-bromo-2'-deoxyuridine; CAF = carcinoma-associated fibroblasts; CK = cytokeratin; DCIS = ductal carcinoma *in situ*; DMEM = Dulbecco's modified Eagle's medium; ECM = extracellular matrix; E:F = ratio of epithelial cells to fibroblasts; ELISA = enzyme-linked immunosorbent assay; FITC = fluorescein isothiocyanate; H&E = hematoxylin and eosin; HPLC = high-performance liquid chromatography; IGF = insulin-like growth factor; NAF = normal breast-associated fibroblasts; PBS = phosphate-buffered saline; PCR = polymerase chain reaction.

## Competing interests

The author(s) declare that they have no competing interests.

## Authors' contributions

AS carried out experimental design and conduct, and manuscript preparation. ZN provided technical assistance with flow cytometry. MRJ provided technical assistance with quantitative real-time PCR, and carried out manuscript preparation. DBB provided technical assistance with the conduction of experiments. SRG provided assistance with the conduction of experiments. GPP provided assistance with the statistical analysis. JVT provided technical assistance and carried out manuscript preparation. DRW carried out experimental design and manuscript preparation. ARF was the principal investigator responsible for overall experimental design and conduct, and manuscript preparation.

## Figures and Tables

**Table 1:** 5-Bromo-2'-deoxyuridine (BrdU) labeling* of MCF10A cells grown in monoculture (control group) and in co-cultures with normal breast-associated fibroblasts (NAF) and carcinoma-associated fibroblasts (CAF)

Culture	BrdU-labeling indices of MCF10A cells (mean ± standard error of the mean)	BrdU-labeling indices of MCF10A cells (group mean ± standard error of the mean)	Comparison of BrdU-labeling indices of MCF10A cells between groups (linear regression)	
MCF10A (*n *= 6)	30.3 ± 3.0	30.3 ± 3.0	MCF10A vs MCF10A + NAF (*P *= 0.009)	MCF10A + NAF vs MCF10A + CAF (*P *= 0.501)
NAF-1 + MCF10A (*n *= 7)	21.9 ± 4.2	16.1 ± 2.6 (*n *= 19)		
NAF-2 + MCF10A (*n *= 6)	10.7 ± 3.6			
NAF-3 + MCF10A (*n *= 6)	14.7 ± 5.0			
CAF-1 + MCF10A (*n *= 8)	15.2 ± 2.0	18.5 ± 2.5 (*n *= 19)	MCF10A vs MCF10A + CAF (*P *= 0.024)	
CAF-2 + MCF10A (*n *= 6)	15.5 ± 4.2			
CAF-3 + MCF10A (*n *= 5)	27.6 ± 6.7			

*Assessed by immunocytochemistry

**Table 2:** 5-Bromo-2'-deoxyuridine (BrdU) labeling* of MCF10AT cells grown in monoculture and in co-cultures with normal breast-associated fibroblasts (NAF) and carcinoma-associated fibroblasts (CAF)

Culture	BrdU-labeling indices of MCF10AT cells (mean ± standard error of the mean)	BrdU-labeling indices of MCF10AT cells (group mean ± standard error of the mean)	Comparison of BrdU-labeling indices of MCF10AT cells between groups (linear regression)	
MCF10AT (*n *= 6)	27.7 ± 7.2	27.7 ± 7.2	MCF10AT vs MCF10AT + NAF (*P *= 0.013)	MCF10AT + NAF vs MCF10AT + CAF (*P *< 0.001)
NAF-1+ MCF10AT (*n *= 7)	17.1 ± 2.8	14.1 ± 1.4 (*n *= 20)		
NAF-2 + MCF10AT (*n *= 6)	13.8 ± 1.9			
NAF-3 + MCF10AT (*n *= 7)	11.4 ± 1.9			
CAF-1 + MCF10AT (*n *= 8)	25.9 ± 4.9	25.5 ± 2.8 (*n *= 22)	MCF10AT vs MCF10AT + CAF (*P *= 0.935)	
CAF-2 + MCF10AT (*n *= 8)	26.6 ± 3.2			
CAF-3 + MCF10AT (*n *= 6)	23.5 ± 7.4			

*Assessed by immunocytochemistry

**Table 3:** Insulin-like growth factor (IGF) II ELISA for normal breast-associated fibroblasts (NAF), carcinoma-associated fibroblasts (CAF), MCF10A cells and MCF10AT cells in monolayer cultures

Cell type	IGF II level (normalized to total protein)	
	
	Level (ng/μg)	Mean (ng/μg)
NAF		
NAF-1	11.63	
NAF-2	6.87	
NAF-3	10.31	
NAF-4	7.21	
NAF-5	2.92	7.79
CAF		
CAF-1	11.80	
CAF-2	10.58	
CAF-3	10.59	
CAF-4	11.27	
CAF-5	10.21	
CAF-6	14.07	11.42
Breast epithelial cells		
MCF10A	5.56	
MCF10AT	4.17	4.87

**Table 4:** Insulin-like growth factor (IGF) II ELISA for normal breast-associated fibroblasts (NAF), carcinoma-associated fibroblasts (CAF) and MCF10AT cell monocultures and co-cultures in a three-dimensional *in vitro *model

Cell type	IGF II level (normalized to total protein)			
	
	Monoculture		Co-culture with MCF10AT cells	
	
	Level (ng/μg)	Mean (ng/μg)	Level (ng/μg)	Mean (ng/μg)
NAF				
NAF-1	49.25		9.68	
NAF-2	45.00		10.22	
NAF-3	68.14	54.13	10.27	10.06
CAF				
CAF-1	70.64		10.01	
CAF-2	52.37		10.88	
CAF-3	52.96	58.65	9.96	10.28
Breast epithelial cells				
MCF10AT	9.98			
